# Improved lag screw positioning in the treatment of proximal femur fractures using a novel computer assisted surgery method: a cadaveric study

**DOI:** 10.1186/1471-2474-15-189

**Published:** 2014-05-30

**Authors:** Matthias Regling, Arno Blau, Robert A Probe, James W Maxey, Brian D Solberg

**Affiliations:** 1 Stryker Trauma GmbH, Schoenkirchen, Germany; 2 Stryker Leibinger GmbH & Co. KG, Freiburg, Germany; 3 Department of Orthopaedics, Scott & White Memorial Hospital, Temple, TX, USA; 4 University of Illinois College of Medicine, Peoria, IL, USA; 5 Department of Orthopaedic Surgery, Keck School of Medicine of the University of Southern California, Los Angeles, CA, USA

**Keywords:** Computer assisted surgery, Lag screw placement, Proximal femur fractures, Hip fractures, Cut-out, Surgical technique

## Abstract

**Background:**

The importance of the tip-apex distance (TAD) to predict the cut-out risk of fixed angle hip implants has been widely discussed in the scientific literature. Intra-operative determination of TAD is difficult and can be hampered by image quality, body habitus, and image projection. The purpose of this paper is to evaluate, through a cadaveric study, a novel computer assisted surgery system (ADAPT), which is intended for intraoperative optimisation of lag screw positioning during antegrade femoral nailing. A 3D measure for optimal lag screw position, the tip-to-head-surface distance (TSD), is introduced.

**Methods:**

45 intra-medullary hip screw procedures were performed by experienced and less experienced surgeons in a cadaveric test series: in 23 surgeries the ADAPT system was used, and in 22 it was not used. The position of the lag screw within the femoral head and neck was evaluated using post-operative CT scans. TAD, TSD, fluoroscopy as well as procedure time and variability were assessed.

**Results:**

The use of the ADAPT system increased accuracy in TSD values (i.e. smaller variability around the target value) for both groups of surgeons (interquartile range (IQR) of experienced surgeons: 4.10 mm (Conventional) vs. 1.35 mm (ADAPT) (p = 0.004)/IQR of less experienced surgeons: 3.60 mm (Conventional) vs. 0.85 mm (ADAPT) (p = 0.002)). The accuracy gain in TAD values did not prove to be significant in the grouped analysis (p = 0.269 for experienced surgeons; p = 0.066 for less experienced surgeons); however, the overall analysis showed a significant increase in accuracy (IQR: 4.50 mm (Conventional) vs. 2.00 mm (ADAPT) (p = 0.042)). The fluoroscopy time was significantly decreased by the use of the ADAPT system with a median value of 29.00 seconds (Conventional) vs. 17.00 seconds (ADAPT) for the less experienced surgeons (p = 0.046). There was no statistically significant impact on the procedure time (p = 0.739).

**Conclusions:**

The ADAPT system improved the position of the lag screw within the femoral head, regardless of the surgeon’s level of clinical experience, and at the same time decreased overall fluoroscopy usage. These positive effects are achieved without increasing procedure time.

## Background

Hip screw cut-out with penetration into the hip joint has been reported to be one of the major complications in the treatment of per-trochanteric hip fractures with fixed angle devices. The occurrence of this complication still ranges from 1.2-8.5% with sliding hip screws and intramedullary nails in recent studies [1-17], although significant improvements in the surgical technique have already led to a decrease in cut-out rates [5]. In earlier studies, its occurrence has been reported to be as high as 12.6-16% [18,19]. Once cut-out has occurred, the patient typically faces difficult reconstructive options, often leaving no other recourse than conversion to total hip replacement [3].

In 1995, Baumgaertner et al. introduced the concept of the tip-apex distance for predicting the risk of failure of fixation by lag screw cut-out [20]. They demonstrated that increasing TAD above 25 mm was strongly correlated with an increased risk of lag screw cut-out through the femoral head. Several other studies supported this conclusion, showing that the TAD is a highly significant predictor of mechanical failure due to cut-out [10,12,14,15,21,22]. In a later study, Pervez et al. recommended a TAD of less than 20 mm [21]. Besides the TAD, the position of the lag screw within the femoral head as described by Parker in 1992 has been identified to influence cut-out [23], with the optimal positioning of the screw remaining controversial. While numerous studies found the centre-centre position in the AP and lateral planes to be most advantageous [18,20,24-26], many authors of both biomechanical as well as clinical studies recommend placing the lag screw in the inferior half of the femoral head in the antero-posterior (AP) view and in the centre of the femoral head in the lateral view [14,17,27-33].

The purpose of the present paper is to introduce and evaluate a computer-assisted surgery (CAS) method that assists the surgeon in accurately positioning the tip of the screw intra-operatively in real time, independent of the position of the lag screw relative to the centre-centre axis of the femoral head. The technique and results of a cadaveric series are presented.

## Methods

### The ADAPT system

**Figure 1:**
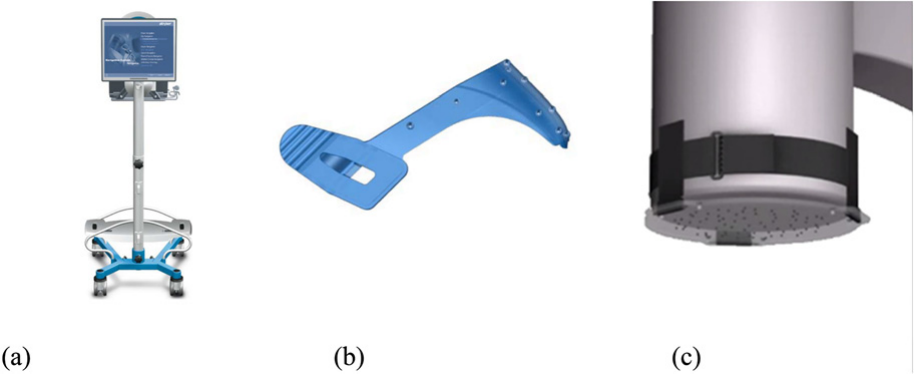
**Special components of the ADAPT system. (a)** Computer Platform, **(b)** ADAPT Clip, **(c)** FluoroDisc.

The ADAPT system (sold and cleared (FDA approved and CE marked) under the name “FluoroMap System”, Stryker Leibinger GmbH & Co. KG, Freiburg, Germany) is a computer assisted stereotaxic device intended to assist a surgeon in the manual surgical placement of the Stryker Gamma3 Trochanteric Nail in proximal femur fracture surgery, with the intention of improving the positioning of the lag screw.The system uses and manipulates 2D fluoroscopic X-ray images taken during the surgical procedure to intraoperatively compute 3D information in real time. This is accomplished using several special components that integrate into the surgical workflow (Figure 1).

**Figure 2:**
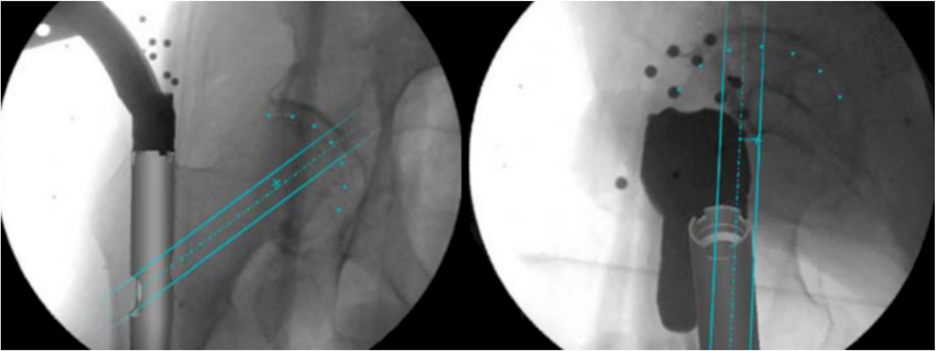
Translational (left) and rotational (right) alignment of the nail.

**Figure 3:**
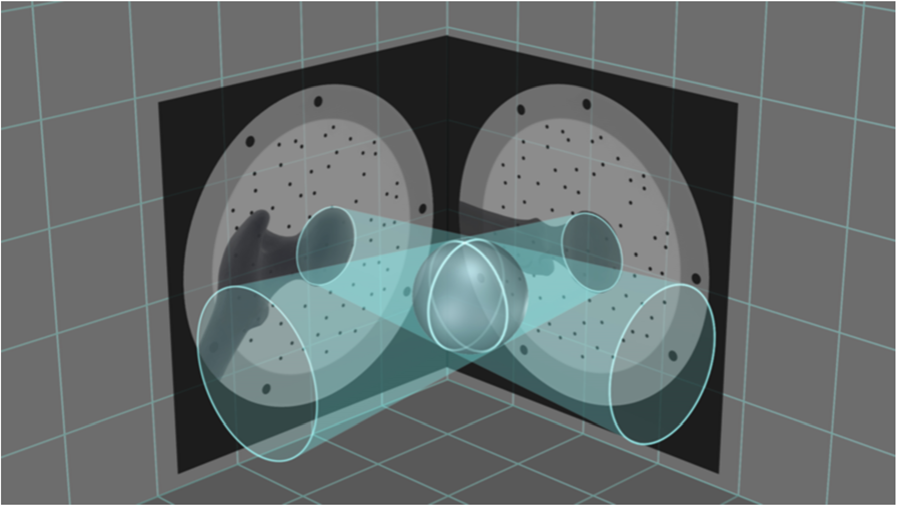
Principle: generation of a 3D model of the anatomy from two 2D X-ray images.

**Figure 4:**
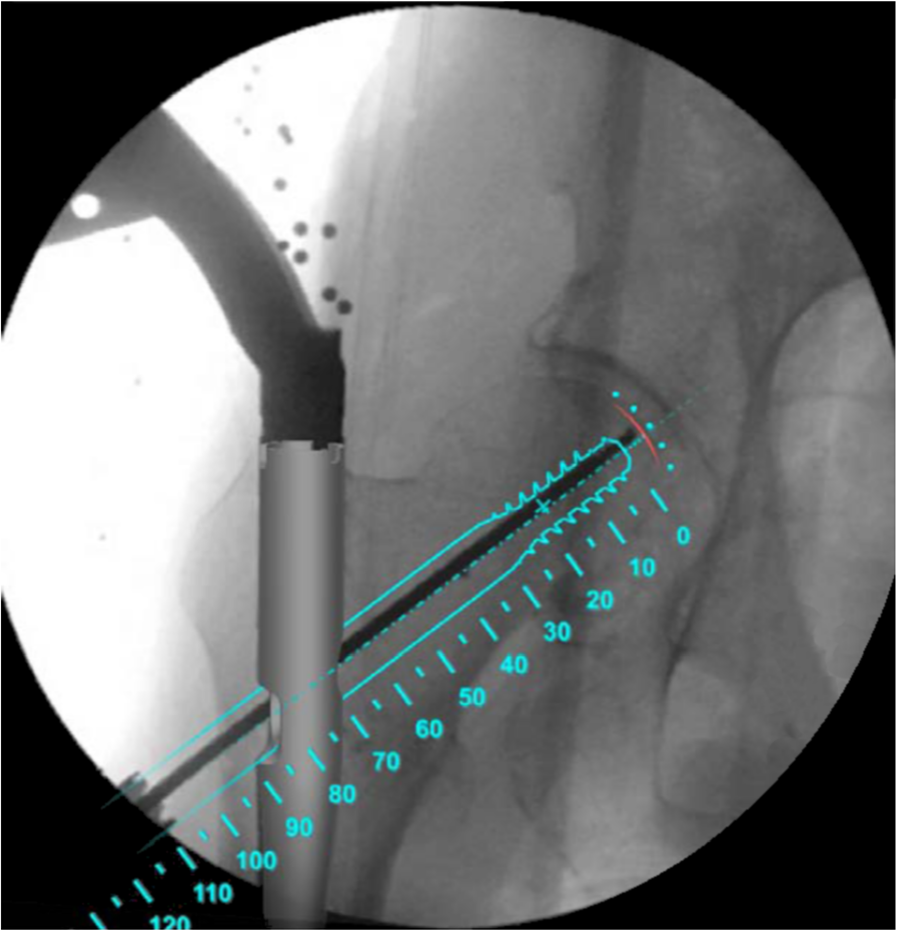
Predicted position of the lag screw and determination of required lag screw length.

**Figure 5:**
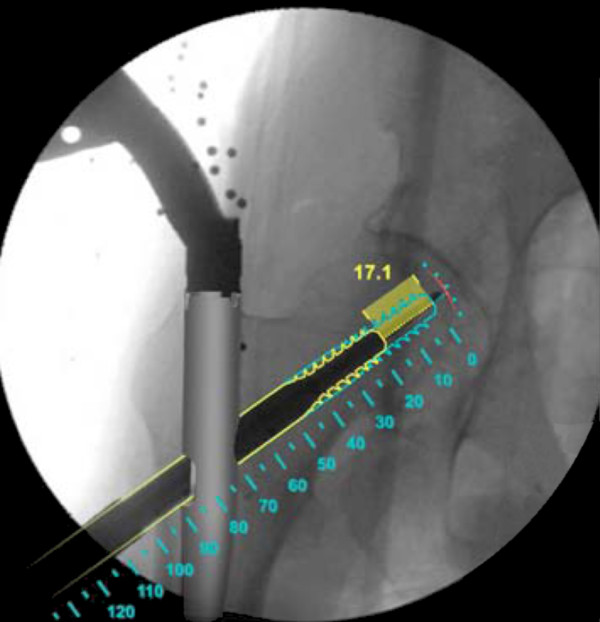
Implantation of the lag screw.

**Figure 6:**
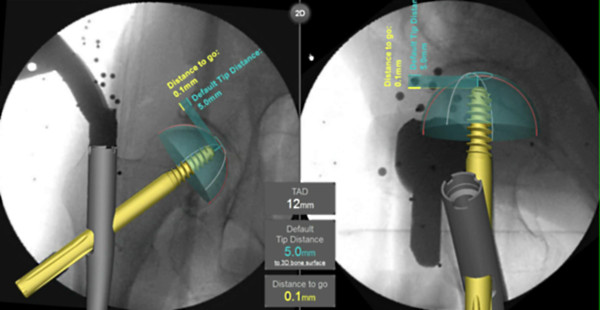
3D model of the position of the implanted lag screw within the femoral head.

The Computer Platform (a) features a display and serves as the platform for the FluoroMap software. A video cable is used for communication and transfer of images between the fluoroscopy unit and the computer platform/software. The ADAPT Clip (b) is made of X-ray translucent materials and contains a defined 3D pattern of metallic marker spheres. It is firmly attached to the Gamma 3 nail targeting device during surgery. The FluoroDisc (c) consists of an X-ray translucent plate containing metallic marker spheres with a known geometrical 2D pattern which is attached to a standard image intensifier of the C-arm using Velcro straps.The FluoroMap software gathers the fluoroscopic images from the C-arm via the video signal. As fluoroscopic images are always distorted due to magnetic fields, the correction of these effects is required before the images can be used for the computation of 3D information. The software achieves this by an automatic detection and utilisation of the metallic marker spheres of the FluoroDisc to dewarp the fluoro images. It automatically segments the femoral head and calculates its centre. Using the known pattern of the metallic marker spheres inside the ADAPT Clip and its projection in the fluoro image, the software computes 3D positional information of the nail and the lag screw to accurately overlay the contour of the implants on the fluoro images. This virtual overlay on the 2D fluoro images assists the surgeon in ideally positioning and aligning the nail by displaying the resulting virtual trajectory of the lag screw prior to placement. AP and lateral spot fluoroscopic images are taken to determine 3D nail position and rotation. The fluoroscopic images do not need to be obtained from precise AP or lateral positions; the only prerequisite is to have a difference of at least 45° between the two images (Figure 2).The software then estimates the appropriate lag screw length to optimise placement and minimise TAD/TSD through the generation of a 3D model of the femoral head (Figure 3).Once the lag screw K-wire is inserted, the software displays the virtual outline of the lag screw as well as a virtual ruler whose tip is positioned at the optimal 3D distance to the femoral head (tip-to-head-surface distance (TSD); see Excursion). This enables the surgeon to read out the required lag screw length at the lateral cortex of the femoral shaft (Figure 4).In order to prevent femoral head perforation during lag screw seating, the software automatically detects the depth of the lag screw and indicates the remaining distance of the tip of the lag screw to the surface of the femoral head every time a new fluoroscopic image is acquired (Figure 5).Once the lag screw is fully seated (actual screw (in yellow) and default screw (in blue) are aligned), the software shows a 3D model of the position of the implanted lag screw within the femoral head. Final TSD and TAD are calculated and visualised to provide the surgeon with information during the surgery (Figure 6).

### Excursion: the difference between the TAD (Tip-Apex Distance) and the TSD (Tip-to-head-Surface Distance)

**Figure 7:**
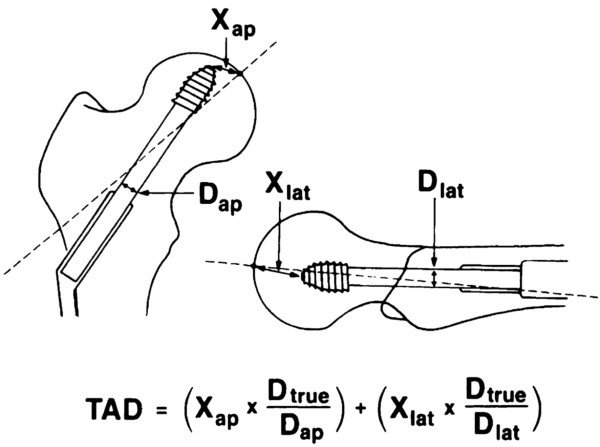
**Measurement of the TAD**[20]**.**

The tip-apex distance (TAD) was defined by Baumgaertner et al. [20]. The TAD is the sum of the 2D distances of the tip of the lag screw to the apex of the femoral head in an anteroposterior and a lateral X-ray image (Figure 7) [20].

The tip-to-head-surface distance (TSD) is a concept for a 3D measurement of the 3D distance of the tip of the lag screw to the surface of the femoral head in direction of the lag screw axis.

**Figure 8:**
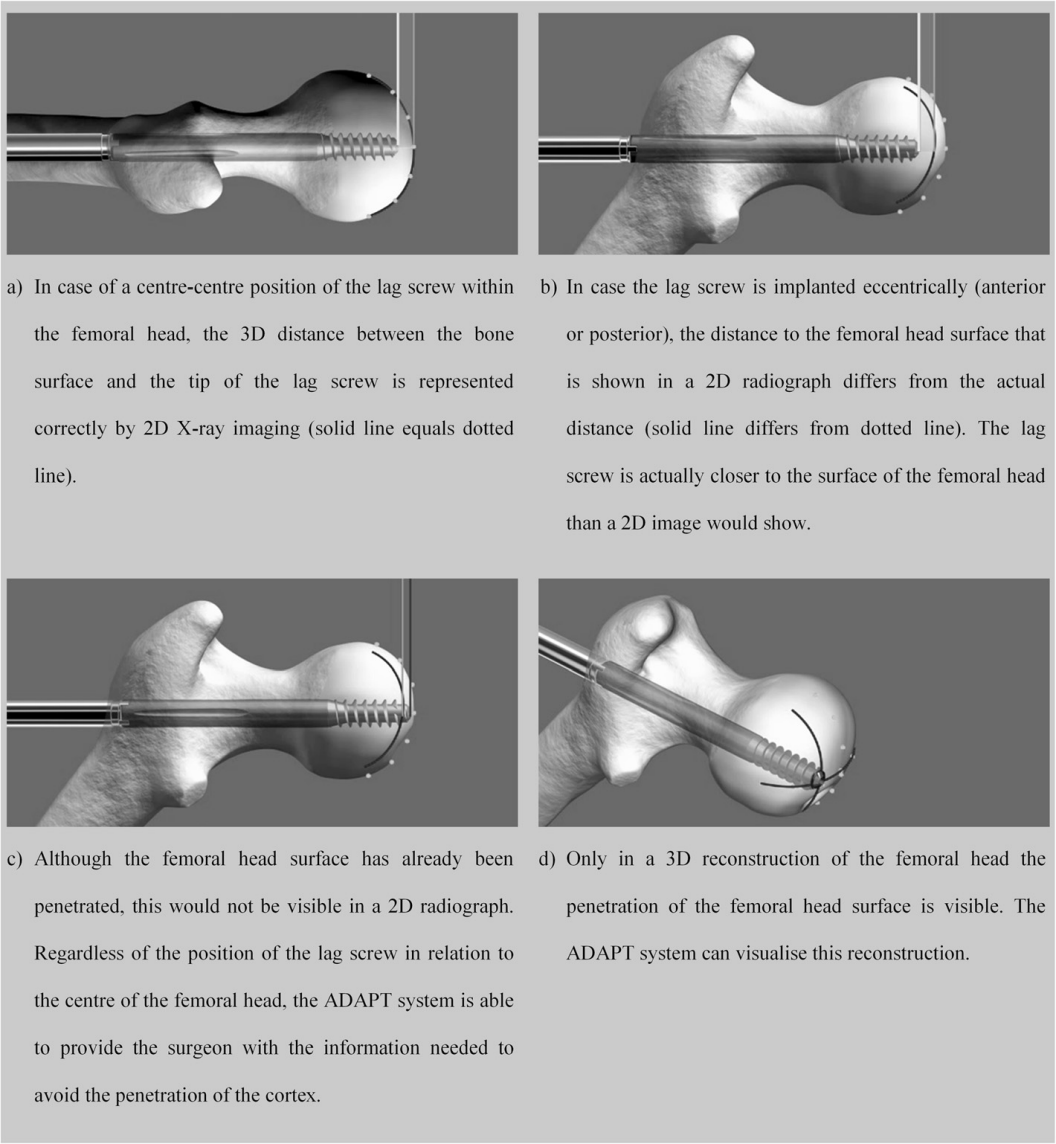
**Difference between 2D and 3D image information; screenshots taken from FluoroMap software (solid lines represent the real surface of the femoral head based on 3D information, dotted lines represent the outline of the femoral head as shown in 2D radiographs). (a)** Congruence of the visible and actual 3D distance to the bone surface in case of a centric lag screw placement, **(b)** lack of congruence of the visible and actual 3D distance to the bone surface in case of an eccentric lag screw placement, **(c)** penetration of the femoral head surface despite inconspicuous 2D imaging, **(d)** 3D reconstruction of the femoral head revealing the penetration of the femoral head surface.

It describes by how much the lag screw could be inserted before penetrating the surface of the femoral head. These two measures, TSD and TAD, can easily be compared. In case of an exact centre-centre position of the lag screw within the femoral head and the identity of the anatomical and implant’s CCD angles a TSD of 5 mm equals a TAD of 10 mm. In reality this exact centre-centre position and CCD angles can hardly be archived during surgery. Therefore, the TAD is often higher than two or three times the TSD value.In case of a deviation of the lag screw axis from the centre-centre position, i.e. an eccentric position of the lag screw, the use of 2D imaging may lead to femoral head perforation despite the appearance of an acceptable TAD. Therefore, an eccentrically positioned lag screw poses the risk of penetrating the joint surface if only 2D information is used (Figure 8).

### Cadaveric test series using the ADAPT system

Two cadaveric tests were conducted to assess the impact of the ADAPT system on the lag screw placement during a Gamma3 surgery. The cadaveric tests were performed at the Texas Health Research & Education Institute in Dallas (TX), USA on September 9th - 10th, 2010, and on April 4th - 5th, 2011 at the Academy for Medical Training and Simulation in Lucerne, Switzerland. Ethics approval was not required in the USA and Switzerland for cadaveric studies as per federal laws. However, the cadaveric test performed in Dallas was approved by the Anatomical Board of the State of Texas and the institute in Lucerne is in full compliance with medical-ethical guidelines and recommendations of the Swiss Academy of Medical Sciences. Consent for the storage and use of the bodies for research purposes was given by all body donors prior to death or by their next of kin.

Experienced surgeons, who perform more than 50 Gamma3 surgeries per year, as well as less experienced surgeons, who perform less than 15 Gamma3 surgeries per year, participated in the cadaveric test series. All surgeons received product training of the ADAPT system, and less experienced surgeons attended a Gamma3 workshop on sawbones.

In total, 45 procedures were performed (Table 1). Three attempted cases were aborted. In one of these cases, a large abdominal mass precluded acceptable fluoroscopic images, and in two cases, unusually dense bone proved difficult to drill with available instruments. The procedures were performed on 12 fresh human cadaveric specimens per test. Each cadaveric specimen had a trochanteric 125 degree Gamma3 nail placed into both right and left proximal femur. The use of the ADAPT system was randomly assigned with equal left and right applications. In the contralateral extremity, the implant was placed using conventional fluoroscopy.

**Table 1:** Case processing summary

	**Experience level**		
**Technique**	**Less experienced**	**Experienced**	**∑**
Conventional	9	13	22
ADAPT	9	14	23
∑	18	27	45

Following this study setup, the participating surgeons were split into four different groups: (1) experienced surgeons using ADAPT, (2) experienced surgeons using conventional fluoroscopy, (3) less experienced surgeons using ADAPT and (4) less experienced surgeons using conventional fluoroscopy.

The surgeons were asked to attempt to position the lag screw in their best estimate of centre-centre position with a distance of 5 mm from the surface of the femoral head (TSD).

The real 3D position of the lag screw within the femoral head was assessed using post-operative CT-scans; no problems with artefacts were encountered during the assessment. The evaluation of the CT-scans was performed with OrthoMap 2.0-19; the reviewer was blinded to sample and method of placement. C-arm images were used to post-operatively measure the TAD. Only the procedural steps that are supported by the ADAPT system were timed (e.g. excluding time needed for patient positioning, incision) and compared to avoid biasing factors. Fluoroscopy times were measured by the C-arm. During analysis and group comparison the statistician was blinded with regard to the identity of the group.

### Statistical analysis

The parameters of interest for the statistical analysis were the accuracy of the lag screw placement as measured with TSD and TAD, the procedure time and the fluoroscopy time. The results were analysed for descriptive statistics with focus on the central position and variation measures. Quantitative data was assessed for normality by using the Shapiro-Wilk test. Due to small subgroup sample sizes and the absence of normality, non-parametric tests were applied for the inferential statistics. In order to assess the differences in accuracy between the groups, the variability of values was analysed by means of the Moses test. Procedure time and fluoroscopy time were examined with the Mann–Whitney test. The robustness of both tests was increased by the application of a Monte Carlo Simulation with 10.000 runs. The significance level for all tests was set at 95% (alpha = 0.05). All statistical analyses were performed using SPSS/PASV V.17.

## Results

### Accuracy - ADAPT vs. conventional technique

#### Tip-to-head-Surface Distance (TSD)

**Figure 9:**
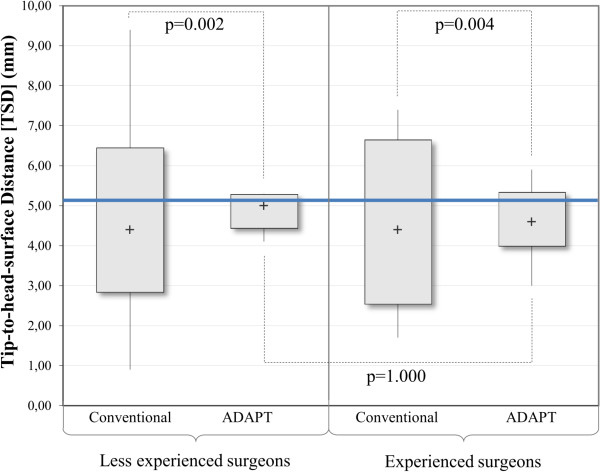
Analysis of the TSD (ADAPT vs. conventional; experienced vs. less experienced), n = 45 (Moses test).

**Figure 10:**
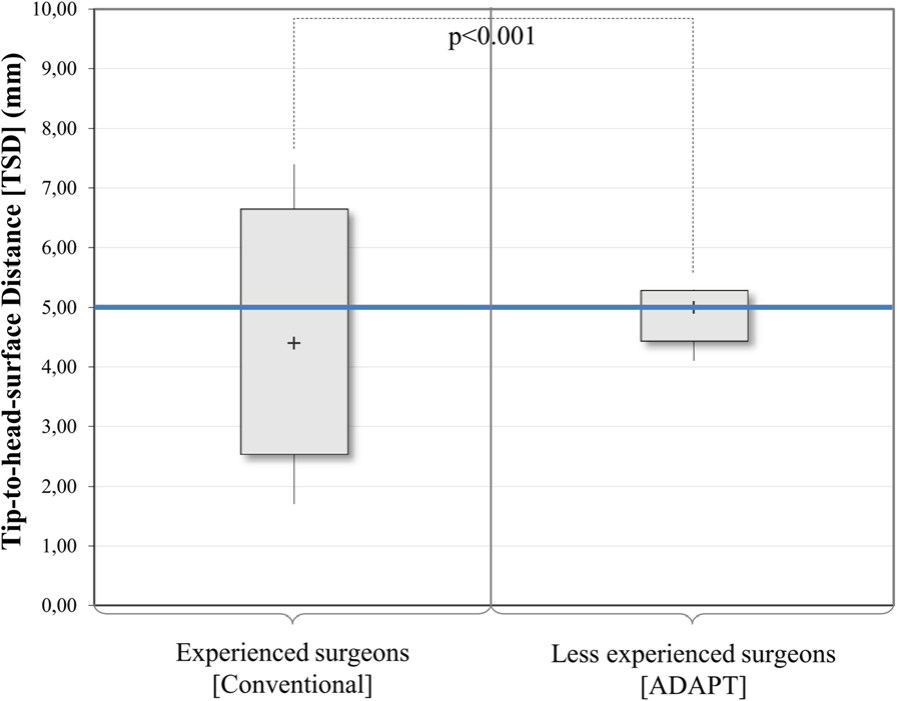
Analysis of the TSD (less experienced [ADAPT] vs. experienced [conventional]), n = 22 (Moses test).

The TSD values for the group of experienced surgeons following the conventional approach (without usage of the ADAPT system) ranged from 1.7 mm to 7.4 mm with a median distance of 4.4 mm. Using the ADAPT system, the TSD ranged from 3.0 mm to 5.9 mm with a median TSD of 4.6 mm. For the less experienced surgeons, the TSD ranged from 0.9 mm to 9.4 mm with a median TSD of 4.4 mm in the conventional cases, whereas it ranged from 4.1 mm to 5.3 mm with the median distance equalling the target value of 5.0 mm in the cases with ADAPT usage (Table 2).The increase in accuracy (i.e. smaller variability around the target TSD) for both groups of surgeons proved to be significant (p = 0.004 for experienced surgeons and p = 0.002 for less experienced surgeons). The inter-group comparison of the cases with the ADAPT system shows that the less experienced surgeons were able to achieve results as accurate as the experienced surgeons for the placement of the lag screw (p = 1.000) (Figure 9).Furthermore, the statistical analysis of the presented data shows that less experienced surgeons using the ADAPT system achieve significantly higher accuracy than experienced surgeons following the conventional approach (p < 0.001) (Figure 10).

**Table 2:** Accuracy TSD - ADAPT vs. conventional technique - descriptive statistics

		**Experience level**		
**Technique**	**Descriptives**	**Less experienced**	**Experienced**	**Overall**
Conventional	n	9	13	22
	Median [mm]	4.40	4.40	4.40
	Range [mm]	8.50	5.70	8.50
	Interquartile range [mm]	3.60	4.10	3.70
ADAPT	n	9	14	23
	Median [mm]	5.00	4.60	5.00
	Range [mm]	1.20	2.90	2.90
	Interquartile range [mm]	0.85	1.35	1.20

#### Tip-apex distance (TAD)

**Figure 11:**
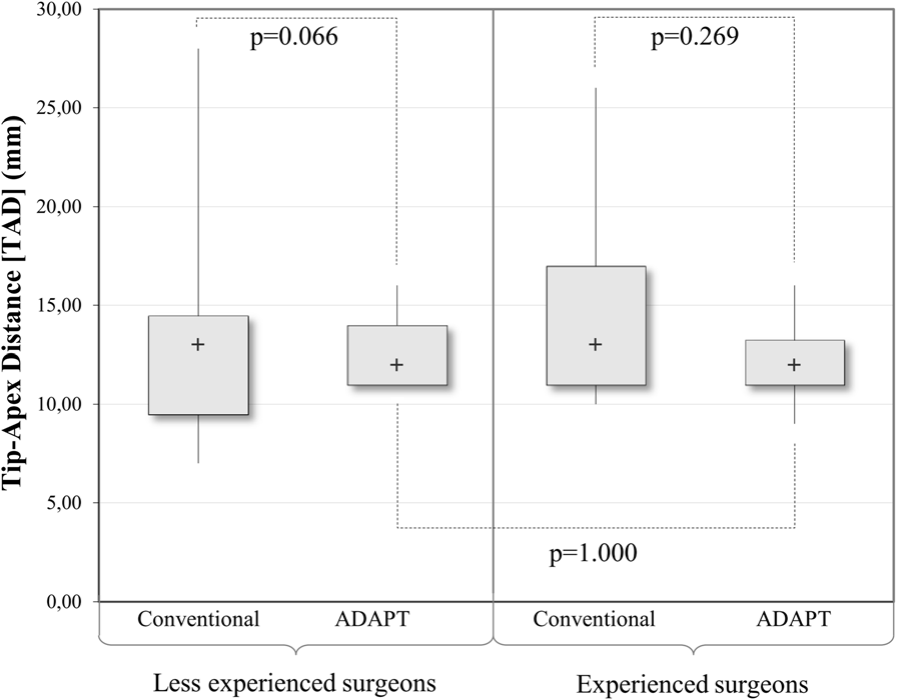
Analysis of the TAD (ADAPT vs. conventional; experienced vs. less experienced), n = 45 (Moses test).

Without the use of the ADAPT system, the TAD values for the experienced group of surgeons ranged from 10 mm to 26 mm with a median distance of 13 mm, whereas it ranged from 9 mm to 16 mm with a median value of 12 mm in the ADAPT cases. For the less experienced surgeons, the TAD ranged from 7 mm to 28 mm with a median TAD of 13 mm in the conventional cases, whereas it ranged from 11 mm to 16 mm with a median distance of 12 mm in the cases supported by the ADAPT system (Table 3). In the ADAPT cases, no TAD exceeded the established threshold of 25 mm nor the reduced threshold of 20 mm, whereas it was greater than 25 mm in two conventional cases.The increase in accuracy (i.e. smaller variability in values) did not prove to be significant for the grouped analysis (p = 0.269 for experienced surgeons and p = 0.066 for less experienced surgeons) (Figure 11); however, the overall analysis shows a significant increase in accuracy in the ADAPT cases (p = 0.042). Moreover, the inter-group comparison of the ADAPT cases shows that the less experienced surgeons were able to achieve results as accurate as the experienced surgeons (p = 1.000).

**Table 3:** Accuracy TAD - ADAPT vs. conventional technique - descriptive statistics

		**Experience level**		
**Technique**	**Descriptives**	**Less experienced**	**Experienced**	**Overall**
Conventional	n	9	13	22
	Median [mm]	13.00	13.00	13.00
	Range [mm]	21.00	16.00	21.00
	Interquartile range [mm]	5.00	6.00	4.50
ADAPT	n	9	14	23
	Median [mm]	12.00	12.00	12.00
	Range [mm]	5.00	7.00	7.00
	Interquartile range [mm]	3.00	2.25	2.00

### Procedure time - ADAPT vs. conventional technique

**Figure 12:**
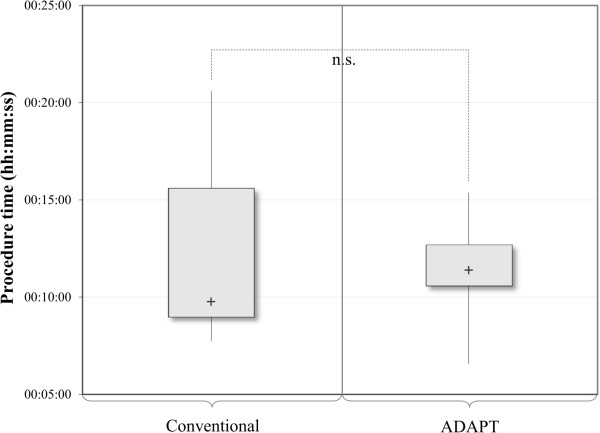
Analysis of the procedure time (ADAPT vs. conventional), n = 18 (Mann–Whitney test).

The procedure time for conventional cases ranged from 7 minutes and 44 seconds to 20 minutes and 37 seconds with a median duration of 9 minutes and 46 seconds, whereas it ranged from 6 minutes and 34 seconds to 15 minutes and 22 seconds with a median time of 11 minutes and 23 seconds for the ADAPT cases (Table 4). There was no significant difference (p = 0.739). Descriptive data and box plots show a tendency for a smaller variation in procedure time for the ADAPT cases, but this could not be confirmed statistically with the current data due to limitation of sample size (Figure 12).

**Table 4:** Procedure time - ADAPT vs. conventional technique - descriptive statistics (only less experienced surgeons)

	**Technique**	
**Descriptives**	**Conventional**	**ADAPT**
n	9	9
Median (hh:mm:ss)	00:09:46	00:11:23
Range (hh:mm:ss)	00:12:53	00:08:48
Interquartile range (hh:mm:ss)	00:06:36	00:02:06

Note: The analysis of procedure time was limited to the group of less experienced surgeons only. The data that was obtained on the cases the group of experienced surgeons performed were not usable due to defects of the OR equipment (C-arm) which influenced the measurements.

### Fluoroscopy time - ADAPT vs. conventional technique

**Figure 13:**
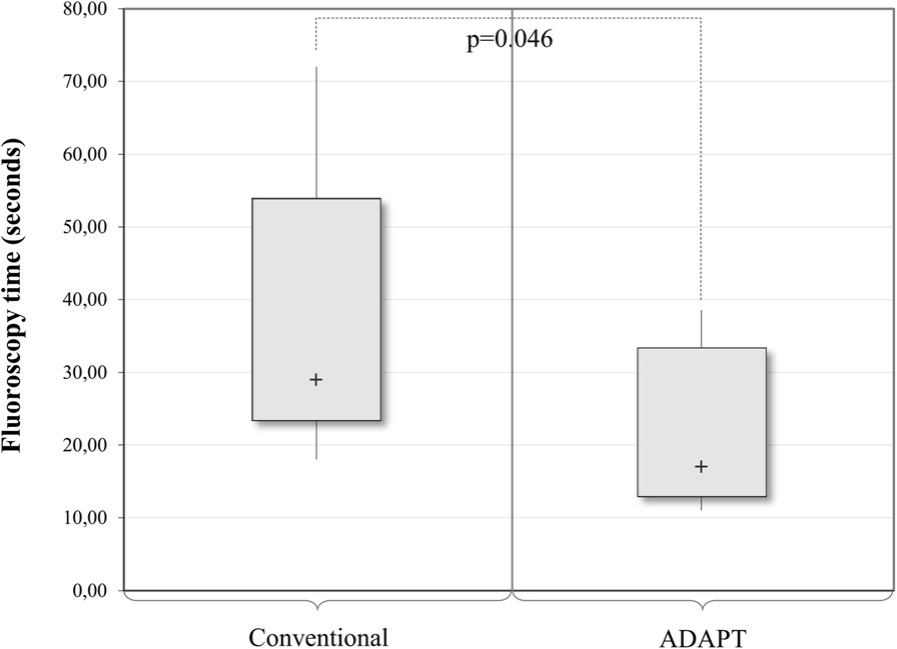
Analysis of the fluoroscopy time (ADAPT vs. conventional), n = 18 (Mann–Whitney test).

The fluoroscopy time for the conventional cases ranged from 18.00 to 72.00 seconds with a median of 29.00 seconds. For the ADAPT cases, the values ranged from 11.00 to 38.00 seconds with a median of 17.00 seconds (Table 5). The decrease in fluoroscopy time in the cases supported by the ADAPT System proved to be significant (p = 0.046) (Figure 13).

**Table 5:** Fluoroscopy time - ADAPT vs. conventional technique - descriptive statistics (only less experienced surgeons)

	**Technique**	
**Descriptives**	**Conventional**	**ADAPT**
n	9	9
Median (seconds)	29.00	17.00
Range (seconds)	54.00	27.00
Interquartile range (seconds)	30.50	20.50

Note: The analysis of fluoroscopy time was limited to the group of less experienced surgeons only. The data that was obtained on the cases the group of experienced surgeons performed were not usable due to defects of the OR equipment (C-arm) which influenced the measurements.

## Discussion

It is widely accepted that the TAD is a highly significant risk predictor of mechanical failure due to cut-out. However, the concept of the TAD has some limitations. Firstly, it is not practical as the TAD is not routinely available intra-operatively. However, assessment of the TAD is required in real time in the operating theatre to serve as an indicator for ideal lag screw placement. Davies et al. suggest using the TAD for a targeted approach to follow up by bringing back those patients with a high TAD for follow-up [34], but the ultimate goal should be to avoid poor lag screw positioning in the first place and to evolve from a retrospective assessment method to an intraoperative quality tool. Available computerized navigation systems improve the accuracy of implant placement [35], but require markers and pre-operative configurations and thus are time-consuming [36]. Atesok and Schemitsch conclude that the proposed advantages of computer-assisted trauma surgery - increased precision, less radiation, and minimised invasiveness - come at the price of major disadvantages, including increased surgical time, a considerable learning curve, cost, as well as special requirements with regards to equipment handling and operating room settings [37]. The ADAPT system provides both TAD and TSD intraoperatively. Our results show that its use does not lead to an increase in surgical time, while accuracy is improved and the radiation exposure is decreased. These effects are achieved with little modification of current surgical and image intensification equipment.

Secondly, the measurement of the TAD can be challenging, especially for inexperienced surgeons. If calculated manually, it is prone to errors and not exact; inter-observer variability was shown to range around 10% [20,34]. Modern picture archiving and communication systems (PACS) meet the requirements for accurate and reproducible measurement of the TAD [38], but again are not practical. The presented system features an automatic and objective calculation of the essential values that serve as a strong predictor of lag screw cut-out in real time, independent of the surgeon’s level of experience. Our data shows that the use of the ADAPT system offers reproducible results.

Thirdly, the TAD concept contains a weakness in focusing on distance and neglecting direction; a recent study found only its AP part to be predictive for failure of fixation [17]. Still, there is no clear consensus about the ideal position of the lag screw in the caudal-cranial direction. A recent biomechanical analysis found an inferior lag screw placement to feature the highest axial and torsional stiffness [33]. De Bruijn et al. recently supported this result with their retrospective study from 2012 on the reliability of predictors for cut-out by identifying the central-inferior and anterior-inferior positions as being highly protective against lag screw cut-out [32]. In another recent study from 2011, Herman et al. defined a “safe zone” for the placement of the lag screw [17]. Implantation of the lag screw outside this zone was shown to be thirteen times more risky in terms of mechanical failure (Odds Ratio 13.4). Remarkably, this “safe zone” was within the inferior half of the femoral head. However, a peripheral lag screw position inherently increases the TAD as the distance to the apex of the femoral head grows [31]. Thus, the explanatory power of the TAD concept diminishes with an eccentric lag screw placement. As shown in the excursus, the minimisation of TAD based on 2D fluoroscopic images during lag screw placement can in extreme cases even lead to articular surface penetration. In contrast, the TSD is a meaningful measure regardless of the relative position of the lag screw within the femoral head. Because it computes the real 3D distance of the tip of the lag screw to the surface of the femoral head, the presented system supports the insertion of the lag screw in all surgical cases, including eccentric placement of the lag screw. Hence, the concept of TSD seems critical for surgeons who choose to place the lag screw in an inferior or non-centre-centre position.

In their study on the characteristics of 57 cut-outs with biomechanical explanation as observed in 3066 consecutive patients treated with Gamma Nails, Bojan et al. identified the combination of three critical factors to drive the risk for mechanical failure due to lag screw cut-out: a complex fracture type, non-anatomical reduction and a non-optimal lag screw position [39]. One individual factor or the combination of two did not explain a cut-out. Hence, by avoiding of non-optimal lag screw position as a contributing factor, a significant reduction in cut-out rates may be achieved.

Awareness of the TAD alone has been shown to reduce the rate of mechanical failure due to an improved position of the lag screw; as a result of increased awareness, the quality of reduction was enhanced [40]. The TAD has been confirmed to be a clinically useful indicator for screw placement. This proven concept can be extrapolated to using real-time TSD measurements. The presented novel system is especially useful for less experienced surgeons as the system enables them to achieve TAD and TSD as accurate as the experienced surgeons. Hence, it may be used ideally for learning purposes as the surgeons get direct feedback in real time on both TAD and TSD.

Our results show that both experienced as well as less experienced surgeons can benefit from the ADAPT system. It seems to be particularly powerful in reducing the variability; lag screws that are placed either extremely close to the cortex or extremely far from the cortex involve a particularly high risk of mechanical failure.

A weak point in our study is that the data on fluoro and procedure times were not usable for the experienced surgeons due to a defect of OR equipment (C-arm) in one of the cadaveric tests that influenced measurements. Further studies should concentrate on these end points. Moreover, our findings relate to a cadaveric setting which results in further limitations: the surgeries in the cadaveric tests were performed on unfractured bones. In clinical cases, complex fracture patterns could impact the surgeon’s ability to accurately position the implants. Furthermore, we did not test the impact of the improved accuracy in terms of an optimized TSD on the strength of fixation as our intent was to study the effect of the ADAPT system on the accuracy of implant placement. However, several studies analysed the correlation of the TAD and the likelihood of a cut-out [10,12,14,15,20-22]. Further biomechanical or clinical studies should be undertaken to investigate whether these findings can be extrapolated to the TSD measurement and whether the TSD proves useful as an intraoperative assessment tool.

## Conclusion

The first experiences with the ADAPT system gained through a cadaveric test series show that both experienced as well as less experienced surgeons can benefit from the ADAPT system through more accurate lag screw placement. The system uses existing equipment and smoothly integrates into the surgical workflow, does not increase procedure time, but statistically decreases the fluoroscopy time. Especially less experienced surgeons can benefit from the system and it can be a useful training tool. However, first experiences are limited to cadaveric tests thus far. Further experiences should be made in clinical settings. Still, the first results seem to be very promising.

## Competing interests

MR is an employee of Stryker Trauma GmbH; AB is an employee of Stryker Leibinger GmbH & Co. KG; RAP, JWM and BDS are consultants to Stryker Orthopaedics. The conduct of the study was financed by various subsidiaries of Stryker as part of verification and validation testing of the system. The article-processing charge is financed by Stryker Trauma GmbH.

## Authors’ contributions

MR was involved in the conception and coordination of the study, contributed to the acquisition and analysis/interpretation of data and drafted the manuscript. AB was involved in the conception and coordination of the study and helped in the data analysis and in drafting the manuscript. RAP, JWM and BDS conceived of the study and revised the manuscript for important intellectual content. All authors read and approved the final manuscript.

## Pre-publication history

The pre-publication history for this paper can be accessed here:

http://www.biomedcentral.com/1471-2474/15/189/prepub
